# Low Molecular Weight Hyaluronan Induces an Inflammatory Response in Ovarian Stromal Cells and Impairs Gamete Development In Vitro

**DOI:** 10.3390/ijms21031036

**Published:** 2020-02-04

**Authors:** Jennifer E. Rowley, Farners Amargant, Luhan T. Zhou, Anna Galligos, Leah E. Simon, Michele T. Pritchard, Francesca E. Duncan

**Affiliations:** 1 Department of Obstetrics and Gynecology, Feinberg School of Medicine, Northwestern University, Chicago, IL 60611, USA; jennifer_rowley@hms.harvard.edu (J.E.R.); farners.amargant@northwestern.edu (F.A.); tracy.zhou@northwestern.edu (L.T.Z.); leahsimon2019@u.northwestern.edu (L.E.S.); 2 Department of Pharmacology, Toxicology and Therapeutics, University of Kansas Medical Center, Kansas City, KS 66160, USA; galligosa@hawks.rockhurst.edu

**Keywords:** hyaluronan fragments, stroma, inflammation, ovarian biology, reproductive aging

## Abstract

The ovarian stroma, the microenvironment in which female gametes grow and mature, becomes inflamed and fibrotic with age. Hyaluronan is a major component of the ovarian extracellular matrix (ECM), and in other aging tissues, accumulation of low molecular weight (LMW) hyaluronan fragments can drive inflammation. Thus, we hypothesized that LMW hyaluronan fragments contribute to female reproductive aging by stimulating an inflammatory response in the ovarian stroma and impairing gamete quality. To test this hypothesis, isolated mouse ovarian stromal cells or secondary stage ovarian follicles were treated with physiologically relevant (10 or 100 μg/mL) concentrations of 200 kDa LMW hyaluronan. In ovarian stromal cells, acute LMW hyaluronan exposure, at both doses, resulted in the secretion of a predominantly type 2 (Th2) inflammatory cytokine profile as revealed by a cytokine antibody array of conditioned media. Additional qPCR analyses of ovarian stromal cells demonstrated a notable up-regulation of the eotaxin receptor *Ccr3* and activation of genes involved in eosinophil recruitment through the IL5-CCR3 signaling pathway. These findings were consistent with an age-dependent increase in ovarian stromal expression of *Ccl11*, a major CCR3 ligand. When ovarian follicles were cultured in 10 or 100 μg/mL LMW hyaluronan for 12 days, gametes with compromised morphology and impaired meiotic competence were produced. In the 100 μg/mL condition, LMW hyaluronan induced premature meiotic resumption, ultimately leading to in vitro aging of the resulting eggs. Further, follicles cultured in this LMW hyaluronan concentration produced significantly less estradiol, suggesting compromised granulosa cell function. Taken together, these data demonstrate that bioactive LMW hyaluronan fragments may contribute to reproductive aging by driving an inflammatory stromal milieu, potentially through eosinophils, and by directly compromising gamete quality through impaired granulosa cell function.

## 1. Introduction

Fibrosis and inflammation are key hallmarks of the aging ovarian stroma [1,2]. The stroma is the extra-follicular sub-compartment of the ovary and is comprised of a heterogeneous mix of cell types including fibroblasts, smooth muscle cells, endothelial cells, theca-interstitial cells, and immune cells in addition to abundant extracellular matrix (ECM) components [3]. Ovarian follicles, the functional units of the ovary consisting of oocytes and their surrounding granulosa cells, develop within the stroma. The stroma provides the mechanical and signaling microenvironment in which female gametes develop. Thus, the age-dependent increase in stromal inflammation and fibrosis likely influences gamete quantity and quality, either directly or indirectly, and resembles aging in other tissues which is associated with a chronic pro-inflammatory state, referred to as “inflammaging” [4,5,6,7,8,9]. This phenomenon contributes to most, if not all, age-related pathologies across organ systems. However, the female reproductive system is unique because it shows overt signs of aging decades before other organs in the human body. Reproductive aging begins in women as early as their mid-thirties and is characterized by a significant decline in oocyte quantity and quality in the ovary, which contributes to adverse reproductive outcomes [2].

Although inflammation likely contributes to fibrosis in the aging ovarian stroma, what initiates and sustains this inflammation is unknown. Endogenous macromolecules and cellular debris, broadly termed damage-associated molecular patterns (DAMPs), initiate and maintain much of this pathogen-free inflammatory milieu in aging tissues [10]. ECM degradation is one major source of DAMPs, which can occur in response to cellular senescence, increased oxidative stress, tissue injury and/or dysregulated waste clearing mechanisms. DAMPs can in turn activate the innate immune system, leading to downstream pro-inflammatory cytokine secretion and immune cell recruitment [10,11,12,13,14]. One of the best characterized pro-inflammatory DAMPs is low molecular weight (LMW) hyaluronan [10,15,16,17].

Hyaluronan is a ubiquitous component of the ECM in mammalian organs, and we recently demonstrated that ovarian hyaluronan is localized to the stromal sub-compartment [18]. Hyaluronan is a glycosaminoglycan comprised of repeating disaccharide units forming a linear polysaccharide chain that is highly poly-disperse in length, ranging in molecular weight from under 10 kDa to over 1.8 MDa [19]. In its high molecular weight form, hyaluronan promotes tissue hydration and homeostasis [20]. As part of normal hyaluronan turnover, larger hyaluronan molecules are depolymerized producing LMW hyaluronan fragments. These fragments are quickly cleared and do not harm surrounding tissues [21]. However, if this process becomes dysregulated, LMW hyaluronan fragments accumulate in tissue and produce an inflammatory response. The inflammatory effects of LMW hyaluronan have been documented in multiple tissues (i.e., the liver, kidney and lung) and in numerous inflammatory and fibrotic diseases [22,23,24,25]. LMW hyaluronan accumulation can occur due to increased reactive oxygen species (ROS) as well as increased abundance or bioactivity of hyaluronan degradation enzymes (hyaluronidases) [22]. Moreover, hyaluronan fragmentation is associated with aging in extra-ovarian tissues, including articular cartilage [26] and skin [27], with both hyaluronidase and ROS implicated in hyaluronan depolymerization. Ovarian antioxidant defenses decrease with advanced reproductive age with a concomitant increase in oxidative damage, which accelerates reproductive aging due to oxidative stress [28]. Because the aging ovarian microenvironment is permissive to HA fragmentation, we hypothesized that bioactive LMW hyaluronan fragments stimulate an inflammatory response in the ovarian stroma in addition to having a direct effect on the follicle.

## 2. Results

### 2.1. Low Molecular Weight Hyaluronan Treatment Induces an Inflammatory Response in Ovarian Stromal Cells In Vitro

**Figure 1:**
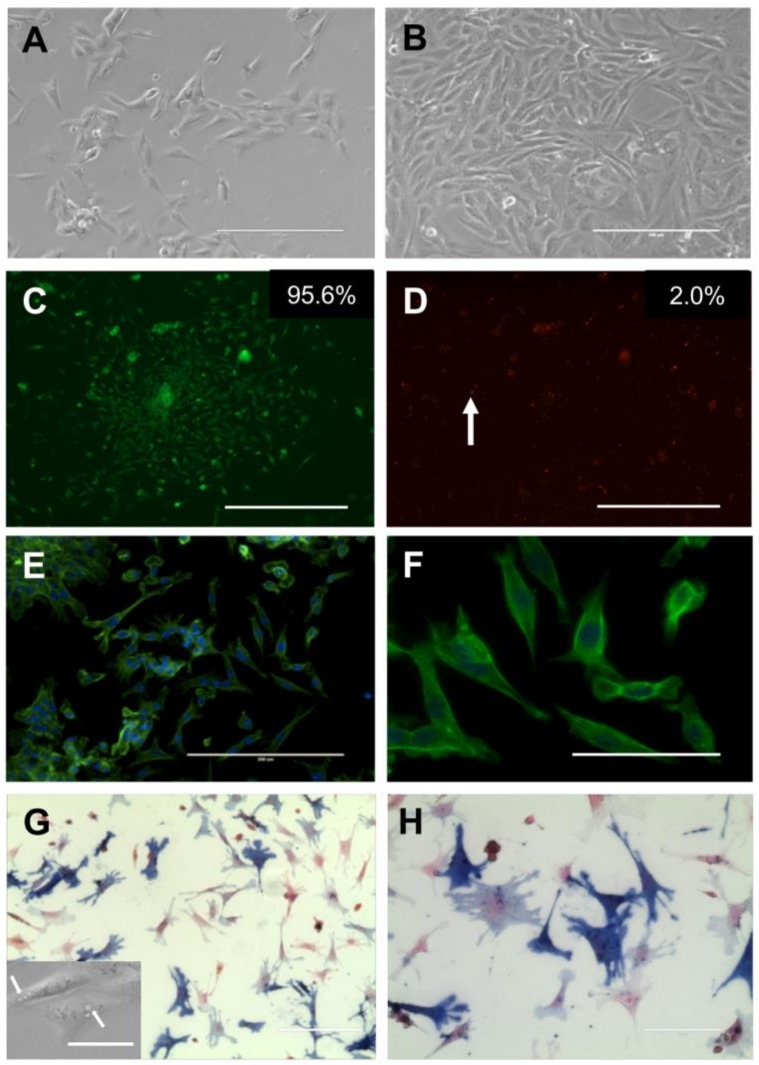
Stromal cell cultures are viable and predominantly theca-like after 48 h. (**A**,**B**) Stromal cells visualized with brightfield microscopy at (**A**) 24 and (**B**) 48 h in culture. Scale bar 200 μm. (**C**,**D**) Representative corresponding images of live cells ((**C**), GFP)) and dead cells ((**D**), TxRed, arrow indicating positive dead stain)). The percentage of total cells staining positively for live and dead stains are shown in insets in (**C**,**D**), respectively. Scale bar 400 μm. (**E**,**F**) Low ((**E**), scale bar 200 μm)) and high (**F**, scale bar 30 μm) magnification images of stromal cells stained for actin with 488-Phalloidin and mounted with 4′,6-diamidino-2-phenylindole (DAPI). (**G**,**H**). Alkaline phosphatase staining (dark blue) to identify cells of theca origin. Low (**G**, scale bar 200 μm) and high magnification ((**H**), scale bar 50 μm)) images. Arrow in G inset (scale bar 30 μm) indicates lipid droplets, a feature associated with theca cells.

Given the known inflammatory properties of LMW hyaluronan fragments in other tissue types, we investigated whether these fragments induced inflammatory cytokine secretion and gene expression in ovarian stromal cells. To do this, we first established and validated a primary ovarian stromal cell culture system. Isolated ovarian stromal cells grew in culture in a monolayer and increased in confluence between 24 and 48 h (Figure 1A,B). After 48 h in culture, 95.6% of cells were alive as assessed by a live/dead staining assay, whereas 2.0% were dead and 2.4% were unstained (Figure 1C,D). The stromal cells were heterogeneous in morphology and cell type as expected [3]. Fibroblast-like cells were detected based on their elongated morphology and presence of multiple F-actin-based stress fibers (Figure 1E,F). Some cells also contained cytoplasmic droplets resembling cholesterol-containing lipid droplets used for steroidogenesis in theca cells (Figure 1G inset) [29]. To determine whether these cells were theca in origin, we performed staining for alkaline phosphatase (AP), which is frequently used as theca marker [3]. After 48 h in culture, 60% of the cells stained positive for AP (Figure 1G,H).

**Figure 2:**
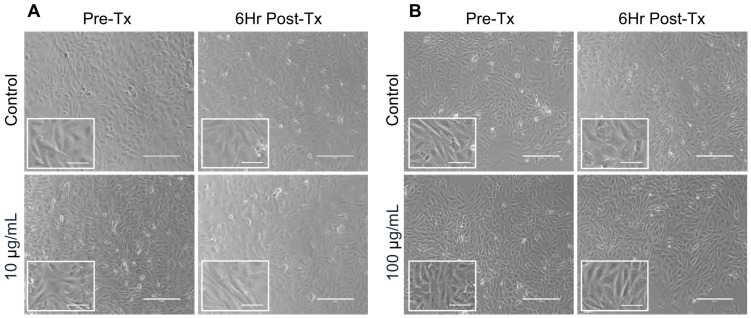
Ovarian stromal cell morphology and confluence is unchanged after 10 or 100 μg/mL low molecular weight hyaluronan treatment. Stromal cells were imaged using brightfield microscopy at 42 h in culture before hyaluronan treatment and after 6 h of hyaluronan treatment at 10 µg/mL (**A**) and 100 µg/mL (**B**). Scale bars 200 µm. Inserts show high magnification brightfield images. Scale bars 75 μm.

**Figure 3:**
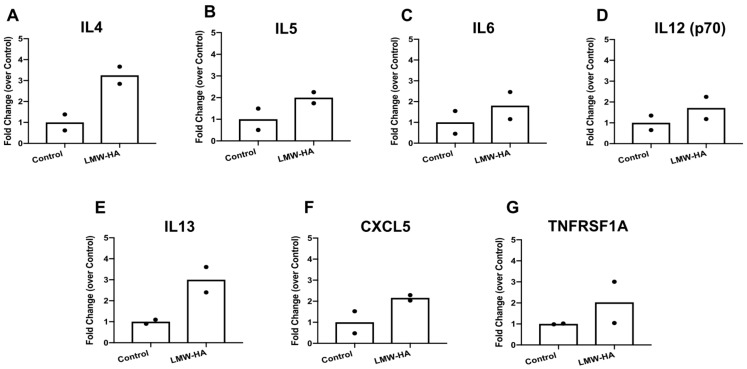
Treatment with 10 μg/mL low molecular weight (LMW) hyaluronan differentially expresses 7 inflammatory cytokines at the protein level. Using a cytokine array, 62 secreted cytokine protein levels were measured in conditioned media after 10 μg/mL LMW hyaluronan treatment. Of the 62 cytokines measured, 7 were differentially expressed: (**A**) IL4, (**B**) IL5, (**C**) IL6, (**D**) IL12 (p70), (**E**) IL13, (**F**) CXCL5, (**G**) TNFSRF1A. The bars represent the average of *n* = 2 replicates. Individual data points are shown.

Next, stromal cell cultures were incubated in the presence or absence of LMW hyaluronan for 6 h. We chose this time point as it was used previously by others [30], and because we wanted to measure cytokine and chemokine proteins secreted by stromal cells into the culture medium. Following a 6 h treatment with 10 or 100 μg/mL 200 kDa LMW hyaluronan, ovarian stromal cells showed no appreciable differences in cell morphology and confluence between the treated and untreated groups, suggesting that these concentrations of hyaluronan were non-toxic and did not trigger differentiation into other cell types (Figure 2). To examine whether LMW hyaluronan induced secretion of pro-inflammatory cytokines, we performed an inflammatory cytokine antibody array on conditioned media from ovarian stromal cells following culture with 10 μg/mL hyaluronan (Figure 3). This array profiles 62 pro- and anti-inflammatory mouse cytokines (Table S1). Despite the short treatment time of 6 h, 7 cytokines showed differential secretion in response to LMW hyaluronan exposure (> 1.45 fold-change): IL4 (3.25 ± 0.58-fold change), IL5 (2.00 ± 0.36-fold change), IL6 (1.81 ± 0.92-fold change), IL12 p70 (1.72 ± 0.75-fold change), IL13 (3.00 ± 0.85-fold change), CXCL5 (2.16 ± 0.18-fold change), and TNFRSF1A (2.02 ± 1.38-fold change) (Figure 3). No cytokines measured were down-regulated (defined as < 0.55 fold-change). Because these data were generated with only two replicates, we did not perform a statistical test for significance.

**Figure 4:**
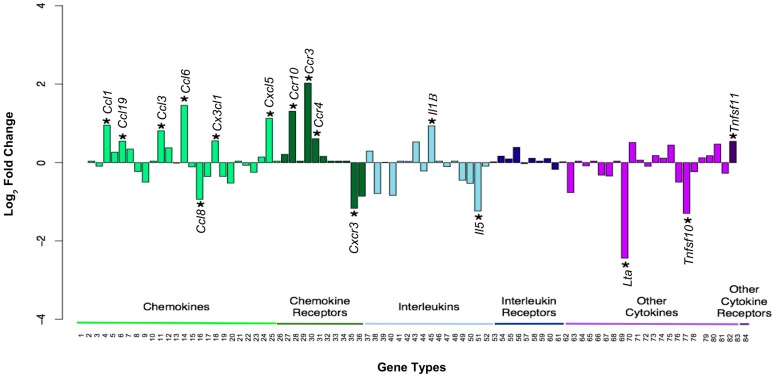
Treatment with 10 μg/mL LMW hyaluronan results in differential expression of inflammatory genes. Gene expression fold changes for all 84 genes were log_2_ transformed to show expression changes relative to zero, and plotted by inflammatory gene type. Gene types include “Chemokines”, “Chemokine Receptors”, “Interleukins”, “Interleukin Receptors”, “Other Cytokines” and “Other Cytokine Receptors”. Up-regulation was defined as a fold change of 1.45 (equivalent to 0.54 on this graph) and down-regulation was defined as 0.55 (equivalent to −0.86 on this graph) were indicated with an asterisk (*).

To further characterize the immune response of ovarian stromal cells following LMW hyaluronan treatment, gene expression patterns in treated versus untreated cells were compared using an inflammatory cytokine and cytokine receptor qPCR array which interrogates the expression of genes encoding select chemokines, inflammatory cytokines and interleukins, as well as their receptors, which mediate inflammation (Table S2). This analysis was performed using the same stromal cells whose conditioned media were analyzed for cytokine and chemokine proteins (Figure 3). Treatment with 10 μg/mL LMW hyaluronan resulted in differential gene expression in sixteen inflammatory genes (> 1.45 or < 0.55 fold-change) (Figure 4, Table 1). Eleven of the sixteen differentially expressed genes encoded for chemokines (*Ccl3*, *Ccl6*, *Ccl8*, *Ccl12*, *Ccl19*, *Cx3cl1*, *Cxcl5*) or chemokine receptors (*Ccr3, Ccr4*, *Ccr10*, *Cxcr3).*

The majority of these eleven chemokine-related genes showed up-regulated expression following treatment relative to controls except for *Ccl8* and *Cxcr3*, which exhibited a down-regulation. The remaining five differentially regulated genes included two interleukins (*Il1β* and *Il5*), as well as three other cytokines and cytokine receptors (*Lta*, *Tnfsf10*, *Tnfrsf11b*). These five genes showed a mix of up-regulation (*Il1β, Tnfrsf11b)* and down-regulation (*Il5*, *Lta*, *Tnfsf10).* We performed a similar gene expression analysis following treatment with 100 μg/mL LMW hyaluronan and found that this higher concentration condition also induced differential patterns of gene expression (Table 1, Supplemental Figure S1).

### 2.2. Genes Involved in IL5-CCR3-Mediated Eosinophil Differentiation, Recruitment, and Maturation Were Differentially Regulated Following LMW Hyaluronan Treatment

**Figure 5:**
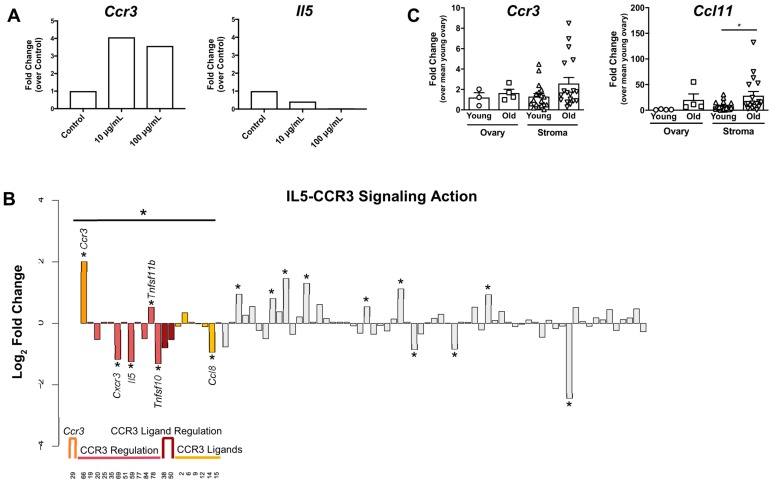
Genes involved in IL5-CCR3-mediated differentiation, recruitment and maturation of eosinophils are differentially expressed LMW hyaluronan treatment in vitro and with age in vivo. (**A**) *Ccr3* and *Il5* expression patterns identified by qPCR array 6 h after 10 or 100 μg/mL LMW hyaluronan treatment *n* = 2. (**B**) A hypergeometric distribution test was performed on 20 of 84 array genes involved in IL5-CCR3-mediated eosinophil activation: *Ccr3* (orange), genes that regulate *Ccr3* expression or CCR3 activity (light red), genes that regulate CCR3 ligand expression or activity (dark red), and genes encoding CCR3 ligands (yellow). Using this test, significantly more genes involved in this pathway were differentially regulated (*, > 1.45 fold-change relative to controls) following LMW hyaluronan treatment than would be expected by chance. (**C**) qPCR analysis was performed using ovaries or ovarian stromal tissue from reproductively young and old mice to compare expression of *Ccr3* and *Ccl11*, a major effector of IL5-CCR3-mediated eosinophil chemotaxis in inflammatory responses. *n* = 3–20. Error bars show standard error of the mean.

Eosinophils are major effector cells of Th2 immunity and are implicated in numerous chronic inflammatory responses [31,32,33]. Interestingly, we observed that *Ccr3* and *Il5,* which are both strongly associated with eosinophil recruitment and differentiation, showed consistent patterns of gene expression across both hyaluronan treatment concentrations. *Ccr3* expression increased in response to LMW hyaluronan relative to controls (10 μg/mL: 4.06-fold and 100 μg/mL: 3.57-fold), whereas *Il5* expression decreased (10 μg/mL: 0.42-fold and 100 μg/mL: 0.02-fold) (Figure 5A). However, at the protein level, IL5 secretion increased following LMW hyaluronan treatment (Figure 3). Twenty of the 84 total genes included in the array are associated with IL5-CCR3 regulation of eosinophils in the context of inflammation. These genes include: CCR3 ligands (*Ccl11*, *Ccl2*, *Ccl24*, *Ccl5*, *Ccl7*, *Ccl8*), regulators of CCR3 ligand expression (*Il4* and *Il13*), regulators of *Ccr3* expression or CCR3 function (*Csf2*, *Cxcl9*, *Cxcl10*, *Cxcl11*, *Cxcr3*, *Ifng*, *Il5*, *Il5ra*, *Tnf*, *Tnfrsf11b*, *Tnfsf10*), and finally *Ccr3* itself. Using a hypergeometric distribution statistical test [34,35], we found that significantly more IL5-CCR3-related genes are differentially regulated after LMW hyaluronan treatment than would be expected by chance (*p* = 0.044) (Figure 5B).

To determine whether these findings may have physiologic significance in the context of reproductive aging, we compared gene expression patterns in whole ovaries and ovarian stromal husks in reproductively old mice versus reproductively young mice. Using qPCR analysis, we observed a consistent trend in the age-dependent increase in *Ccr3* expression in both the ovarian stroma (2.58 ± 2.48-fold change over young whole ovary, *p* = 0.0588) and the whole ovary (1.65 ± 0.73-fold change over young whole ovary, *p* = 0.491), consistent with our in vitro results (Figure 5C). Further, *Ccl11*, the major ligand for *Ccr3* which selectively regulates eosinophil trafficking in inflammatory contexts [36,37], showed a significant increase in reproductively old stroma (28.32 ± 34.11-fold change over young whole ovary, *p* = 0.0151). In the reproductively old whole ovary, the increase in *Ccl11* expression was not significant (20.05 ± 23.52-fold change over young whole ovary, *p* = 0.1590) (Figure 5C).

### 2.3. Low Molecular Weight Hyaluronan Does not Compromise Follicle Growth or Survival but Does Reduce Estradiol Production during eIVFG

**Figure 6:**
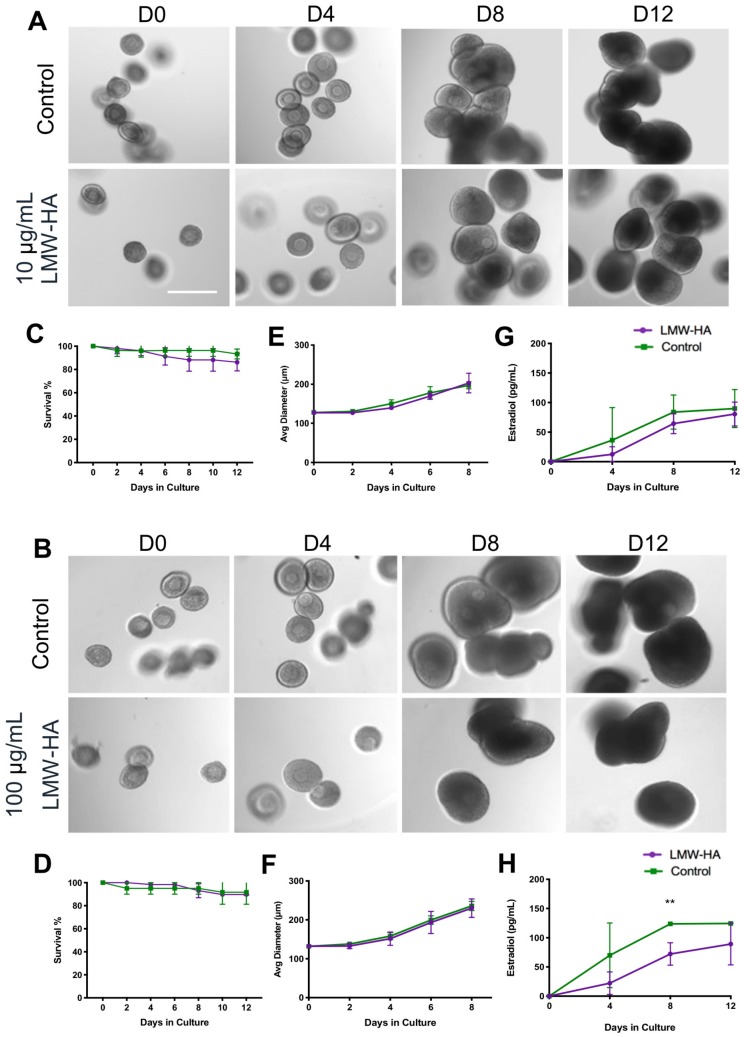
Treatment with 10 or 100 μg/mL LMW hyaluronan does not impact follicle morphology, survival or growth but does reduce estradiol production in an encapsulated in vitro growth system. Three or more follicle cultures were performed with both 10 μg/mL (*n*= 102 treated and 54 control follicles) and 100 μg/mL (*n*= 74 treated and 40 control follicles) 200 kDa LMW hyaluronan (LMW-HA) treatment conditions. Representative images for the 10 μg/mL and 100 μg/mL cultures on day 0, 4, 8 and 12 are shown (**A** and **B**, respectively; scale bars 200 μm). Diameters and survival were tracked every other day throughout culture, with survival and growth curves shown for 10 μg/mL (**C**,**E**) and 100 μg/mL (**D**,**F**) cultures. Estradiol was measured using an ELISA in conditioned follicle culture media on day 0, 4, 8 and 12 for 10 μg/mL (**G**) and 100 μg/mL (**H**) cultures. Control follicle survival, growth and estradiol curves are shown in green and treated follicle survival, growth and estradiol curves are shown in purple. Error bars show standard deviation. ** *p* = 0.0098.

Within the ovary, follicles themselves may be targets of LMW hyaluronan in addition to the stroma. To determine the direct effect of LMW on the ovarian follicle during folliculogenesis, we used an encapsulated in vitro follicle growth (eIVFG) system. Secondary stage follicles were cultured in alginate beads, and follicle morphology, survival, and growth were tracked over 12 days. Treatment with either LMW hyaluronan concentration (10 or 100 μg/mL) did not affect gross follicle morphology or follicle survival and growth relative to controls (Figure 6A,B). At both concentrations, follicle survival was above 80% in the treatment and control groups at day 12 (Figure 6C,D). On day 0 of culture, follicles of equal diameter were placed into treatment and control conditions (10 μg/mL: 127.7 ± 4.2 μm treatment versus 127.4 ± 4.9 μm control; 100 μg/mL: 129.5 ± 7.3 μm treatment versus 131.9 ± 7.8 μm control) (Figure 6E,F). By day 8 of culture, 10 μg/mL treated follicles had an average diameter of 202.9 ± 25.1 μm compared to controls with an average diameter of 197.2 ± 9.2 μm (*p* = 0.681). Similarly, in the 100 μg/mL condition, day 8 follicles had grown to an average of 230.0 ± 23.8 μm in the treated group and 235.9 ± 11.9 μm in the control group (*p* = 0.720). Thus, follicle growth was not affected by LMW hyaluronan treatment, resulting in growth curves that were indistinguishable between experimental and control groups (Figure 6E,F). Furthermore, follicles in all experimental groups reached the large antral stage at the end of the culture period.

To investigate how LMW hyaluronan exposure impacted the function of granulosa cells, 17β-estradiol concentrations were measured in the conditioned media [38]. Following treatment with 10 μg/mL LMW hyaluronan, follicles consistently produced less estradiol than controls on day 0, 4, 8 or day 12, although this was not statistically significant (Figure 6G). Similarly, follicles treated with 100 μg/mL LMW hyaluronan produced less estradiol on every day of culture, and this was significant on day 8 of culture (72.2 ± 19.1 pg/mL treated vs. 123.8 ± 2.7 pg/mL control, *p* = 0.0098) (Figure 6H).

### 2.4. Low Molecular Weight Hyaluronan Treatment of Follicles Compromises Gamete Quality and Causes Premature Resumption of Meiosis

**Figure 7:**
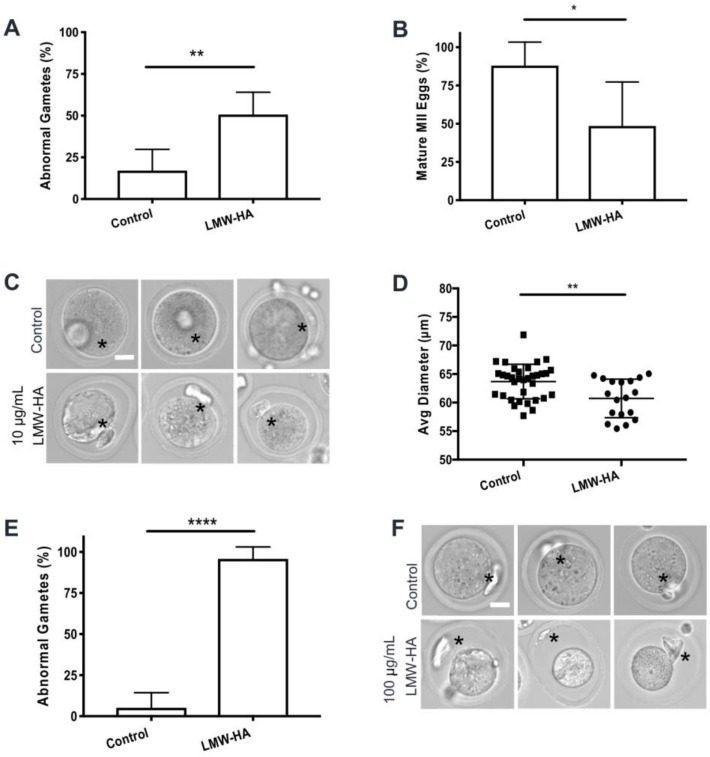
Treatment of follicles with 10 μg/mL and 100 μg/mL LMW hyaluronan in vitro compromises gamete quality. (**A**) Percent of “normal” or “abnormal” gametes after 10 μg/mL LMW hyaluronan treatment (*n* = 71) or control (*n* = 46) post hCG-induced ovulation (** *p* = 0.0035, two-sample t-test). (**B**) Percent of mature metaphase II (MII) eggs (morphologically normal ovulated gametes with extruded polar body) (*n* = 34 10 μg/mL LMW-HA treated follicles, *n* = 38 control follicles). (* *p* = 0.0213, two sample t-test). (**C**) Representative brightfield microscopy images of MII eggs from 10 μg/mL LMW hyaluronan treated and control follicles. Asterisks (*) indicate polar bodies. Scale bar 20 μm. (**D**) Mature MII egg diameters from treated (*n* = 18) and untreated (*n* = 34) follicles (** *p* = 0.0023, two-sample t-test). (**E**) Percent “normal” or “abnormal” gametes after 100 μg/mL LMW hyaluronan treatment (*n* = 35) or control (*n* = 34) after hCG-induced ovulation (**** *p* < 0.0001, two-sample t-test). (**F**) Representative brightfield microscopy images of MII eggs from 100 μg/mL LMW hyaluronan treated and control follicles. Asterisks (*) indicate polar bodies. Scale bar 20 μm. Error bars show standard deviation.

While follicle growth, survival and morphology were unaffected by LMW hyaluronan treatment, a reduction in estradiol is indicative of reduced granulosa cell function, which may impact gamete quality. Thus, we next investigated morphology and meiotic competence in gametes derived from follicles treated with LMW hyaluronan. In the 10 μg/mL LMW hyaluronan condition, follicles produced a significantly higher proportion of morphologically abnormal gametes relative to controls (50.7% versus 17.1%, respectively, *p* = 0.0035) (Figure 7A). When further examining the subset of only morphologically normal gametes, only 48.1% of gametes derived from 10 μg/mL LMW hyaluronan treated follicles reached metaphase of meiosis II (MII) following human chorionic gonadotropin (hCG) exposure relative to 90.0% of controls (*p* = 0.0213) (Figure 7B). In addition, MII eggs from 10 μg/mL LMW hyaluronan treated follicles had significantly smaller terminal diameters than controls (60.7 ± 3.4 μm vs. 63.7 ± 3.0 μm respectively, *p* = 0.0023) (Figure 7C,D).

**Figure 8:**
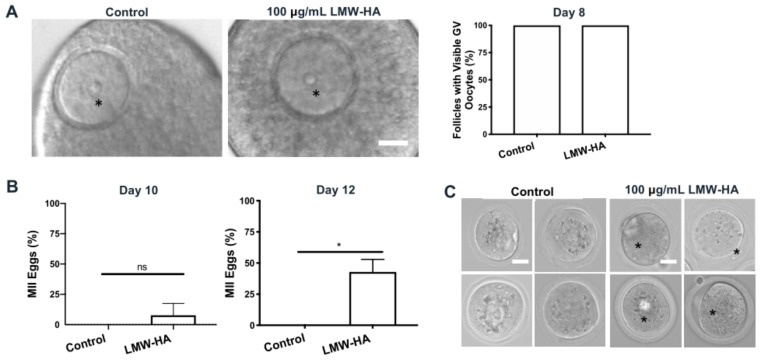
Follicle exposure to 100 µg/mL LMW hyaluronan, *in vitro*, triggers premature meiotic resumption in the oocyte. (**A**) Representative images of oocytes arrested at prophase I (intact germinal vesicles (GV, asterisks) are evidence of meiotic arrest) at day 8 in 100 μg/mL LMW hyaluronan treated (*n* = 75) and control (*n* = 43) follicles (left). Scale bar 20 μm. Percent of follicles with visible GV oocytes on day 8 (right). (**B**) Percent MII eggs on day 10 (*n* = 75 versus *n* = 43 control follicles) and day 12 (*n* = 16 versus *n* = 22 control follicles) post 100 μg/mL LMW-HA treatment, without hCG treatment (ns; not significant, *p* = 0.089, * *p* = 0.026) (two-sample t-test). Error bars show standard deviation. (**C**) Representative brightfield microscopy images of mature MII eggs harvested from 100 μg/mL LMW hyaluronan treated follicles without hCG-triggered meiotic resumption. Asterisks (*) indicates polar bodies. Scale bars 20 μm.

In the 100 μg/mL LMW hyaluronan condition, this impaired gamete phenotype was more severe than the 10 μg/mL condition. Treated follicles produced a significantly higher proportion of abnormal gametes compared to controls (97.4% vs. 8.8%, respectively, *p* < 0.0001) (Figure 7E). In fact, all but one gamete from 100 μg/mL LMW hyaluronan-treated follicles (*n* = 35 total, 34 abnormal) was classified as normal, which precluded evaluation of meiotic endpoints. Although the gametes derived from the 100 μg/mL LMW hyaluronan treated follicles had severely retracted and darkened cytoplasm, 70% of the cells possessed what appeared to be polar bodies, suggesting that they had prematurely resumed meiotic maturation and arrested at MII while still within the follicle and then undergone in vitro aging (Figure 7F). Therefore, we examined the meiotic stage of gametes within the context of intact follicles on days 8, 10 and 12 of culture without induction of ovulation (Supplemental Figure S2). At day 8 of culture, germinal vesicles (GVs), indicative of prophase I arrest, were uniformly visible across all oocytes in 100 μg/mL treated and control follicles as expected (Figure 8A). However, on day 10 of culture, 6.7% of follicles treated with LMW hyaluronan contained mature MII eggs, and this increased to 37.5% by day 12, which was significant relative to untreated controls in which all oocytes were arrested at prophase I (*p* = 0.026) (Figure 8B). The MII eggs collected from follicles at days 10 and 12 had normal morphology with minimal perivitelline space, clear cytoplasm without visible inclusions and round polar bodies still in contact with the egg (Figure 8C).

## 3. Discussion

Using two controlled in vitro systems, we have demonstrated that physiologically relevant concentrations of LMW hyaluronan exert an adverse effect on both major compartments of the ovary (i.e., the stroma and follicles). In ovarian stromal cells, a short-term treatment with 10 and 100 μg/mL LMW hyaluronan resulted in differential expression and secretion of numerous pro-inflammatory cytokines at the mRNA and protein levels, respectively. This was most notable for genes related to eosinophil activation. Further, while these same concentrations of LMW hyaluronan did not significantly impact folliculogenesis endpoints (i.e., follicle growth, morphology, survival), follicles exposed to these concentrations of LMW hyaluronan secreted less estradiol and produced gametes with compromised morphology and meiotic competence, suggesting impaired granulosa cell function. A significant portion of gametes produced from 100 μg/mL LMW hyaluronan-exposed follicles prematurely resumed meiosis. These findings suggest that LMW hyaluronan may be a potential driver of age-associated inflammation in the ovarian stroma. Further, these data have important implications for our understanding of how endogenous sources of tissue damage leading to hyaluronan fragmentation might contribute to the reproductive aging phenotype in both major compartments of the ovary.

In general, the ECM changes throughout the normal aging process. These changes include alterations in composition, increased macromolecule crosslinking, and ECM protein fragmentation [39]. For example, overall hyaluronan content is reduced and is often associated with a concomitant shift to lower molecular weight hyaluronan species in an age-related fashion. These observations are well-documented in articular cartilage [26] as well as in skin [27,40,41] and are paralleled, at least in part, by ultraviolet light-induced accelerated skin aging [42]. Thus, hyaluronan loss is a hallmark feature of aged tissues. The mechanisms by which hyaluronan is lost could involve reduced synthesis and increased degradation, with both enzymatic and non-enzymatic hyaluronan depolymerization proposed. Moreover, increased hyaluronan degradation products can enhance pro-inflammatory signaling and further accelerate aging-related tissue changes. Thus, LMW hyaluronan species could be one of the molecular drivers of the chronic inflammation and fibrosis observed in ovaries from reproductively old mice [1] and this is currently under investigation.

Although we do not know the identity of all cell types in our heterogeneous stromal cultures, previously published work demonstrated that immune cells are maintained [3]. Thus, we anticipate that the responses we observed were at least partly due to immune cell modulation. We have shown that hyaluronan localizes to other cell types in the ovarian stroma, such as the theca layer, so it is possible that LMW hyaluronan could be eliciting responses in other cell types as well as in immune cells [18]. Nevertheless, our stromal gene expression analyses demonstrated that LMW hyaluronan induces largely Th2-biased inflammation, and this was paralleled by secretion of a largely Th2 cytokine profile, including IL4, IL5, IL6 and IL13.

It is interesting to note that the stromal cell LMW hyaluronan response was not entirely regulated by dose. This could be because we explored gene expression at only one time point (6 h) after treatment. Alternatively, it is possible that the two doses elicit completely different biological pathways which is supported by the differential patterns of gene expression observed. Regardless, upon antigenic stimulation, tissue resident immune cells produce these effector cytokines to stimulate an innate immune response, typically against parasites and allergens [43]. In our system, LMW hyaluronan specifically activated genes in ovarian stromal cells that are typically responsible for the differentiation, recruitment, and maturation of eosinophils, a major effector cell of Th2 inflammatory responses, including increased *Ccr3* gene expression and increased IL5 secretion. Although consistent with an eosinophil-related signature, it is worth noting that changes in stromal cell transcript accumulation likely represent both direct LMW hyaluronan-induced changes as well as indirect changes due to cytokines, chemokines, and/or other molecules induced by LMW hyaluronan earlier in the culture period. In other systems, LMW hyaluronan has been shown to directly modulate eosinophil activation. Specifically, LMW hyaluronan treatment of eosinophils from asthma patients significantly increased their survival [44]. This effect is partially mediated by hyaluronan’s primary receptor, CD44, which is expressed on the surface of eosinophils. Further, hyaluronan fragment signaling through CD44 in eosinophils induces maturation [45,46].

**Figure 9:**
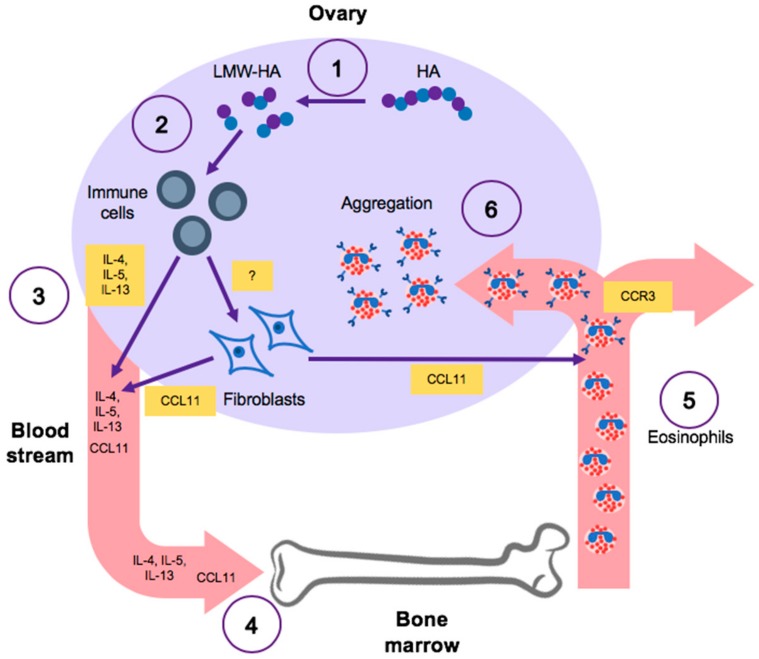
Schematic of potential model of LMW hyaluronan-driven inflammation in the stroma. (**1**) With age, the ovarian stroma likely becomes more permissive of HA fragmentation into LMW hyaluronan (LMW-HA) due to increased enzymatic (hyaluronidase) and/or non-enzymatic (ROS species) mechanisms. This leads to an accumulation of bioactive LMW hyaluronan in the ovarian stroma. (**2**) LMW-HA binds to receptors on tissue resident immune cells in the ovarian stroma, which in turn triggers inflammatory signaling. (**3**) Ovarian tissue resident immune cells mount a systemic Th2 inflammatory response by releasing Th2 pro-inflammatory cytokines (IL4, IL5, IL13) into the blood stream. It is possible that these same immune cells secrete factors that could stimulate fibroblasts in the ovarian stroma to release chemokines capable of promoting immune cell recruitment into the tissue and stimulating production of immune cells in the bone marrow, such as CCL11. (**4**) Secreted cytokines and chemokines trigger production of innate immune cells, such as eosinophils, in the bone marrow. (**5**) Innate immune cells are released into blood stream and undergo subsequent maturation by expressing key chemokine receptors, such as CCR3, which facilitate their recruitment into ovarian tissue. (**6**) Mature eosinophils are recruited into ovarian stroma in response to ovarian stromal LMW hyaluronan.

Our findings suggest that LMW hyaluronan may induce Th2 inflammatory signaling in tissue-resident immune cells in the ovarian stroma that together exert an eosinophilic response (Figure 9) [47]. LMW hyaluronan may accomplish this by dysregulating expression of specific pathways, such as the IL5-CCR3 signaling cascade, that are involved in eosinophil differentiation, recruitment, and maturation. In response to inflammatory stimuli in a tissue, IL5 is released, by local sentinel immune cells into the blood stream which stimulates production of eosinophils by committed bone marrow progenitor cells [31]. Eosinophils released from the bone marrow then home to peripheral tissue undergoing an inflammatory response by upregulating CCR3, as this receptor is vital for responding to several chemotactic factors [48]. Expression of CCR3 on eosinophils is stimulated by cytokines released from immune cells, particularly IL5 [47,49,50,51]. Our proposed model is corroborated by our in vivo observations in the aging ovarian stroma, which revealed a nearly 28-fold expression change in *Ccl11*, the gene that encodes the potent chemoattractant CCL11, which serves as the primary ligand of CCR3 [36]. This model would account for the significant up-regulation in *Ccr3* expression in vitro and *Ccl11* expression in vivo. Although supported by the present data, this model is currently speculative and under further investigation. However, a recently published study corroborates this model by showing a significant increase in eosinophils when comparing ovarian leukocyte populations in 3-month-old vs. 15-month-old mice [52].

Our results suggest a potential role for eosinophils in driving age-related chronic inflammation in the ovary. While few studies have examined eosinophils in ovarian immune function and dysfunction, several clinical reports have documented cases of eosinophilic oophoritis [33,53,54]. These reports confirm that in humans, excess eosinophil recruitment in the ovary can cause significant and dysregulated inflammation, resulting in tissue damage. However, there is also evidence that eosinophils facilitate some ovarian functions under homeostatic conditions. For example, eosinophils assist with corpus luteum regression [55,56]. In other tissues, while eosinophils are best known for their responsiveness to allergens and parasites, these immune cells also recognize and respond to numerous endogenous DAMPs, including LMW hyaluronan [56]. Excessive endogenous DAMPs can also dysregulate signaling pathways that mediate eosinophil function, cytotoxicity, and abundance in tissue—a phenomenon implicated in several chronic inflammatory and fibrotic pathologies, such as asthma and eosinophilic esophagitis [33].

While LMW hyaluronan accumulation may drive reproductive aging by inducing stromal inflammation, our data also suggest that it may directly contribute to a reduction in granulosa cell function, resulting in a decline in gamete quality. Previous studies from our group have demonstrated that follicles from reproductively old mice assume a striking inflammatory pattern of gene expression [57]. It is possible that endogenous sources of inflammation, such as LMW hyaluronan, may be triggering these pathways in the follicle, and this warrants further investigation.

In this study, we used an eIVFG system to investigate whether LMW hyaluronan impacts the follicle compartment of the ovary. Preceding ovulation, dormant follicles in the ovary are activated and undergo extensive growth and development during folliculogenesis. Across several species, the eIVFG system has been used to successfully mature early stage follicles by maintaining vital oocyte-granulosa cell interactions over the course of long-term culture and has ultimately resulted in the production of fertilizable oocytes [58,59,60]. We demonstrated that follicles cultured in an eIVFG system in physiologically relevant levels of LMW hyaluronan displayed impaired readouts of granulosa function across two measures: estradiol production and maintenance of meiotic arrest, and these readouts were dose dependent. In coordination with theca cells, granulosa cells in growing follicles produce estrogens which help regulate the menstrual cycle. In previous eIVFG-based studies, estradiol levels in conditioned media were used as a marker of granulosa cell function [61,62]. LMW hyaluronan exposure reduced estradiol production independent of follicle growth, survival and morphology, and this reduction was significant late in culture. The observation that a significant difference is not reached until later in the culture is not surprising given that it takes time for estradiol to accumulate in the conditioned media. Our findings are likely biologically relevant because the mean estradiol levels are consistently lower in follicles treated with both doses of LMW hyaluronan at all time points compared to untreated follicles. Furthermore, the reduction in estradiol in response to LMW hyaluronan occurs coordinately with a premature resumption of oocyte meiosis within the context of an intact follicle. These phenotypes are both consistent with compromised granulosa cell function.

Meiosis in mammalian oocytes begins during embryonic development and arrests in late prophase of meiosis I. Oocytes remain at this meiotic stage until the LH surge signals mature follicles to resume meiosis and ovulate. Granulosa cells play a critical role in maintaining meiotic arrest of oocytes in prophase I, which can be sustained for upwards of 50 years in the human [63]. In the preovulatory follicle, meiotic arrest is in part dependent on inhibitory molecules, such as cyclic GMP (cGMP) which is produced by granulosa cells and diffuses into the oocyte through gap junctions [64]. LH binding to the outermost granulosa cells in the follicle leads to broad reduction of cGMP across all granulosa cells and in turn the oocyte, thus allowing for meiotic resumption in coordination with ovulation [65,66]. If granulosa cell function is altered, their ability to maintain meiotic arrest in the oocyte may be compromised. For example, resumption of meiosis can occur from blocking signaling between the oocyte and somatic cells [67,68]. It is also possible that meiotic resumption can occur with increased signaling through meiotic activation pathways, such as the MAPK/ERK1/2 pathway [67,68]. The MAPK/ERK1/2 pathway is indispensable for germinal vesicle breakdown (GVBD) and meiotic resumption in oocytes in mammals [68]. Interestingly, the two major hyaluronan receptors CD44 and hyaluronan-mediated motility receptor (RHAMM) are potent signaling molecules which can stimulate MAPK/ERK1/2 signaling pathways following hyaluronan binding [69]. Interestingly, LMW hyaluronan can increase signaling through MAPK/ERK1/2 via the RHAMM receptor compared to HMW hyaluronan [70]. Numerous studies have shown that signaling through these receptors impacts meiotic resumption of oocytes in pigs, blastocyst formation and quality in mice, and enhanced trophoblast growth and invasion in humans [71,72,73]. Thus, LMW hyaluronan may stimulate MAPK/ERK1/2 pathway signaling in granulosa cells and thus drive premature meiotic resumption in the oocyte. Whether the result of reduced granulosa cell inhibition or directly activated meiotic resumption pathways, the significance of dysregulated timing of meiotic resumption can impact fertility. In the fallopian tube, mature eggs undergo degeneration if not fertilized. Ostensibly, mature eggs would similarly undergo degeneration if they resumed meiosis too early while still within the follicle as we observed in follicles exposed to LMW hyaluronan.

One caveat of this study is that we did not consider controls beyond omission of LMW hyaluronan in the treatment of stromal cells or follicles. Indeed, additional studies could be performed to compare 200 kDa LMW hyaluronan with high molecular weight (HMW) hyaluronan (e.g., 1 MDa). Although it would be conceptually satisfying to perform parallel studies with HMW-HA, we do not know its stability especially in culture, and it would be difficult to interpret results due to a heterogeneous composition of fragment sizes. Another potential control would be degradation of 200 kDa LMW hyaluronan to tetra or disaccharides prior to treating stromal cells or follicles. However, these molecules could exhibit their own unique effects in ovary-derived in vitro culture systems. Equally as important as these controls would be a study identifying which hyaluronan receptor(s) are required for the observed biological effects reported here. For example, given previous work demonstrating critical importance of TLR4, CD44 and RHAMM in hyaluronan-mediated biology/pathobiology, each of these receptors could be interrogated; this is the subject of future studies.

Our exclusive use of two tightly controlled in vitro systems provided a unique opportunity to dissect the impact of LMW hyaluronan on the stromal and follicular ovarian sub-compartments independent of one another. However, this approach does have drawbacks. As mentioned above, we neither know the identity of all cell types found in the stromal cultures nor which of these cells are responding to LMW hyaluronan. Moreover, the possibility exists that an integrated response is occurring over the 6 h culture period which could be further dissected by including shorter time points after LMW hyaluronan treatment. However, the impact LMW hyaluronan has on follicles contained in the eIVFG system is follicle intrinsic as this system does not contain immune or other stromal cells, allowing a precise evaluation of LMW hyaluronan in this sub-compartment. Comparing all these data to those found in whole ovaries from young and old mice could corroborate the current findings, and these studies are ongoing.

Together, our findings indicate that potential products of an aging ECM can have a dual impact on both the ovarian stroma and follicles via direct or indirect mechanisms. Although restricted to in vitro systems, these results provide support implicating hyaluronan degradation products in age-related inflammation and fibrosis associated with reproductive decline in mice [1]. Moreover, this work is consistent with previously published studies that document abundant LMW hyaluronan in aging articular cartilage and skin [26,27]. Whether accumulation of LMW hyaluronan fragments occurs in ovarian tissue with advanced reproductive age is an active area of investigation. If this hypothesis is realized, the mechanisms by which ovarian LMW hyaluronan fragments accumulate could be due to an imbalance in hyaluronan synthesis and its degradation. Given the ovary’s rapid aging relative to other organs, studying age-related DAMPs such as LMW hyaluronan in the ovarian stroma may offer novel insights into physiological changes that contribute to a chronic inflammatory and fibrotic aging phenotype in other organs. Further, to our knowledge, our findings constitute the first observations to suggest that that DAMPs produced with aging might directly impact gamete quality.

## 4. Materials and Methods

### 4.1. Animals

Reproductively young adult CB6F1 female mice (aged 3–4 weeks) were purchased from Envigo (Madison, WI, USA) and used for experiments at 6–12 weeks of age. Reproductively old CB6F1 female mice (aged 14–17 months) were procured from the Aged Rodent Colony from the National Institute of Aging. To obtain pre-pubertal mice, CB6F1 hybrids were generated by breeding Balb/c females and C57BL/6 males purchased from Envigo. Pre-pubertal mice were used at 12–14 days of age to maximize the yield of early stage secondary follicles. All mice were housed in a controlled barrier facility at Northwestern University’s Center for Comparative Medicine (Chicago, IL, USA). Temperature, humidity and photoperiod (14 light:10 dark) were kept constant. Animals were provided food and water ad libitum. Breeder mice were fed Teklad Global irradiated chow (2920), and all other mice were fed Teklad Global irradiated chow (2916), which contain minimal phytoestrogens (Madison, WI, USA). All experiments involving animals were performed under protocols approved by the Northwestern University Institutional Animal Care and Use Committee (approved 8/30/2017) and were in accordance with the National Institutes of Health Guide for the Care and Use of Laboratory Animals.

### 4.2. Ovarian Stromal Cell Isolation

Ovarian stromal cell isolation and culture techniques were performed based on previously described methods [3]. In brief, ovaries from 6–12 week-old CB6F1 mice were removed from the bursa and placed in dissection media composed of Leibovitz’s L-15 Medium (L15) (Life Technologies, Grand Island, NY, USA) supplemented with 0.5% Penicillin-Streptomycin (Pen-Strep) (Life Technologies, Grand Island, NY, USA), and 1% fetal bovine serum (FBS) (Life Technologies, Grand Island, NY, USA). To enrich for stromal cells, ovaries were each poked approximately 100 times with two insulin needles to release granulosa cells and oocytes. The remaining stromal husks were cut into four pieces and placed into pre-equilibrated enzymatic media comprised of αMEM Glutamax (Life Technologies, Grand Island, NY, USA) supplemented with 0.5% Pen-Strep and 1% FBS, 0.7 mg/mL Collagenase IV (Thermo Fisher Scientific, Waltham, MA, USA) and 0.2 mg/mL DNAse I (Thermo Fisher Scientific, Waltham, MA, USA). The stromal husks were digested at 37 °C in a humidified environment of 5% CO_2_ in air for one hour. Every 15 min, the tissue was briefly removed from the incubator and triturated 30 times to assist with the digestion process. After 1 h, the enzymatic digestion was quenched using an equal volume of equilibrated media with 10% FBS. The cell-containing mixture was strained through a 40 μm cell strainer to remove undigested ECM and tissue (Greiner Bio-One, Kremsmünster, Austria). Cells were pelleted (1490× *g* for 5 min at room temperature) and washed using 5 mL of 37 °C plating media (RPMI 1640 containing 25 mM HEPES and 2 mM L-glutamine (Invitrogen, Carlsbad, CA, USA) supplemented with 10% FBS and 1% Pen-Strep), three times. Cells were re-suspended in 2 mL plating media. A small volume of suspension (10 μL) was used to count live cells using Trypan Blue Exclusion (Cat. #T10282, Thermo Fisher Scientific, Waltham, MA, USA) and a hemacytometer. After counting, cells were plated in 4-well plates at a density of 33,000 cells/cm^2^. Cells were cultured for a total of 48 h to minimize major changes in cell populations [3]. After 24 h of culture (37 °C, 95% air/5% CO_2_), wells were washed 3 times with warm PBS to remove all non-adhered cells and then imaged using brightfield microscopy on an EVOS FL Auto microscope (Life Technologies, Grand Island, NY, USA) at 4×, 10×, 20× and 40× magnifications.

### 4.3. Low Molecular Weight Hyaluronan Treatment of Stromal Cell Cultures

Eighteen hours after imaging stromal cells as described above, cells were washed 3 times with warmed PBS and media was replaced with fresh media containing 10 or 100 μg/mL 200 kDa LMW hyaluronan (Cat. #HA200K, Lifecore Biomedicals, Chaska, MN, USA), or fresh media without LMW hyaluronan. Although the in vivo hyaluronan concentrations in the ovary are unknown, the selected concentrations are physiologically relevant in other organs [74]. All experiments were performed with 200 kDa LMW hyaluronan, since this size has inflammatory properties in other tissues [16,75,76]. Hyaluronan was certified endotoxin and protein-contamination free by the manufacturer. All treatment times were 6 h in length. Experiments were performed in triplicate for each treatment concentration, with duplicate wells for each condition within each experiment.

### 4.4. Cell Viability Assay

To assess stromal cell viability after 48 h in culture, we used a commercial Live/Dead Assay (Cat. #ab115347, Abcam, Cambridge, MA, USA) according to the manufacturer’s protocol. Live/dead staining was visualized using an EVOS FL Auto microscope with a 20X objective (live cells: green fluorescence protein (GFP) channel (470 nm excitation; 510/42 nm emission), dead cells: Texas Red (TxRed) channel (585/29 nm excitation; 624/40 nm emission). Images in each channel were taken in 5 random regions around each well. Using the EVOS cell counting function, live cell signals were automatically counted. Dead cell signals were counted manually. Using the corresponding transmitted light images, any cells without either live or dead staining were identified. Total cell count was calculated by adding live cells, dead cells and unstained cells. Viability was reported as the average number of live cells over the total number of cells. Each assay was performed in triplicate.

### 4.5. Alkaline Phosphatase and Actin Cytoskeleton Staining

Alkaline phosphatase (AP) is a marker for theca cells, an ovarian stromal cell subtype that is associated with growing follicles and produces the androgens that granulosa cells convert into estrogens [77]. To assess the proportion of our stromal cell population that was comprised of theca cells, AP staining was performed as previously described [3]. In brief, stromal cells adhered to the bottom of the well plate were washed three times with PBS and fixed for 5 min in 4% paraformaldehyde (Electron Microscopy Sciences, Hatfield, PA, USA). Cells were washed three more times in PBS. Cells were then incubated in a histochemical stain (0.25 mg/mL naphtol AS/BI phosphate and 0.75 mg/mL fast blue salt prepared in 0.2 M Tris–HCl, pH 8.3) for 45 min. Cells were washed three times with double-deionized H_2_O (dd-H_2_O) and co-stained with Nuclear Fast Red (0.5%) for 10 min. After three additional washes with dd-H_2_O, cells were imaged in 5 random regions of each well using brightfield microscopy with an EVOS FL Auto and 10X and 40X objectives. The number of cells that were AP-positive out of the total number of cells were quantified and the results were corroborated by a blinded third party. Each assay was performed in triplicate.

To visualize actin-based stress fibers, we stained F-actin using Alexa Fluor 488-Phalloidin (Life Technologies, Grand Island, NY, USA). To do this, stromal cells were cultured on Poly-D-Lysine (Thermo Fisher Scientific, Waltham, MA, USA) coated cover slips (concentration 200 μg/mL). Cells were washed three times with warm PBS and fixed for 5 min in 4% paraformaldehyde (Electron Microscopy Sciences, Hatfield, PA, USA). Cells were washed three more times in PBS and then incubated with a 1:50 dilution of Alexa Fluor 488-Phalloidin for 1 h. After washing three more times with warm PBS, coverslips were mounted on slides with Vectashield containing DAPI (Vector Laboratories, Burlingame, CA, USA) to co-stain DNA. Slides were imaged using an EVOS FL Auto equipped with 10X and 40X objectives.

### 4.6. Cytokine Antibody Array of Ovarian Stromal Cell-Conditioned Media

To examine whether LMW hyaluronan induced an inflammatory response detectable at the protein level, conditioned media were collected from ovarian stromal cell cultures at the conclusion of the 6-h treatment period. All media (0.75 mL per well) were immediately snap frozen. Media were then thawed and pooled, where 0.25 mL of conditioned media from each replicate was combined and diluted with blocking buffer provided with the cytokine antibody array kit in a 1:1 dilution. Samples were then applied to a RayBio C-Series Mouse Cytokine Antibody Array C3 according to the manufacturer’s protocol (RayBiotech, Norcross, GA, USA) and as described previously [1]. Fold change between untreated and treated samples were then calculated. We were unable to determine statistical significance as we only had two replicates per experimental condition, and this was required to ensure we had enough biological material to perform the protein arrays.

### 4.7. Gene Expression Analysis of Ovarian Stromal Cells Using Pathway-Targeted Inflammatory Cytokine and Cytokine Receptors PCR Arrays

Inflammatory gene expression changes in ovarian stromal cells were examined following LMW hyaluronan treatment. Total RNA was extracted and isolated from cultured cells using the RNeasy Mini Kit (Cat. #74104, Qiagen, Valencia, CA, USA) as described previously [78]. To achieve the minimum cDNA concentration required for the inflammatory gene array (see below), we reduced the volume of RNase-free water added to our cDNA mixture to 17.4 μL (10 μg/mL treated and control samples) or 36 μL (100 μg/mL treated and control samples). This adjustment was pre-approved by the manufacturer.

The cDNA was then pooled by treatment group and transcripts analyzed using the Mouse Inflammatory Cytokine and Cytokine Receptor RT^2^ Profiler PCR Array (Cat. #PAMM-011Z, Qiagen, Valencia, CA, USA) according to the manufacturer’s protocol. This array profiles the expression of 84 key genes mediating the inflammatory response, including chemokines, cytokines, interleukins and their receptors. A CFX384 (BioRad, Hercules, CA, USA) machine was used for the real time polymerase chain reaction (PCR). The 5 housekeeping genes included in the array (*Actb*, *B2m*, *Gapdh*, *Gusb*, *Hsp90ab1*) were used to normalize gene expression. Using the 2^−ΔΔCt^ method, fold changes in expression were calculated for each gene in treated over control samples. Statistical significance cannot be determined because the data represent only two replicates per experimental group.

### 4.8. Gene Expression Analysis of Ovarian Tissue and Ovarian Stromal Husks by qPCR

To examine age-related changes in eosinophil-associated gene expression in the ovarian stroma in vivo, we examined *Ccr3* and *Ccl11* expression in whole ovaries as well as from stromal husks from reproductively young and old mice. Stromal husks were prepared from 20 young mice and 19 old mice as described above and were then placed in RNAlater Stabilizing Solution (Cat. #AM7020, Thermo Fisher Scientific, Waltham, MA, USA). Whole ovaries were harvested from 4 young and 4 old mice. A bead homogenization system (FastPrep 24, MP Biomedicals) was used to homogenize stromal husks and whole ovaries in RLT buffer containing β-mercaptoethanol (RNeasy Mini Kit, Qiagen). RNA was then isolated from tissue lysates using the RNeasy Mini Kit as described above and 1 μg RNA was reverse transcribed to cDNA using a High Capacity cDNA Reverse Transcription Kit (Cat. #4368813, Thermo Fisher Scientific, Waltham, MA, USA). Real time PCR was then performed using Power SYBR Green (Invitrogen, Carlsbad, CA, USA) in a CFX384 machine (BioRad, Hercules, CA, USA). After normalizing to 18S as a house keeping gene, fold changes in transcript content were calculated relative to the mean young whole ovary value, using the 2^−ΔΔCt^ method.

### 4.9. Follicle Isolation and Encapsulated In Vitro Follicle Growth (eIVFG)

The effect of LMW hyaluronan treatment on follicle growth and survival was assessed using an established eIVFG system for studying folliculogenesis, in vitro [61,79]. Ovaries were isolated from 12–14 day-old CB6F1 female mice and placed in dissection media, and follicles were mechanically isolated. Early secondary follicles were selected based on size (between 120–140 μm) and morphology (intact basement membrane, visible and healthy oocyte, two layers of granulosa cells) and placed into maintenance media (αMEM Glutamax supplemented with 0.5% Pen-Strep and 1% FBS). To encapsulate follicles, groups of 10 follicles were pipetted into the center of 0.5% (w/v) alginate drops (7.5 μL). The alginate drops containing follicles were suspended and gently dropped into a crosslinking solution (50 mM CaCl_2_ and 140 mM NaCl) for 2 min to solidify the hydrogel. Alginate beads were transferred to a 96-well plate with a single bead placed in each well containing 100 μL of equilibrated growth media (αMEM Glutamax, 3 mg/mL BSA (MP Biomedicals, Solon, OH, USA), 10 mIU/mL follicle stimulating hormone (FSH), 1 mg/mL fetuin, 0.1% insulin-transferrin-selenium (Thermo Fisher Scientific, Waltham, MA, USA)).

To assess the effect of LMW hyaluronan treatment on follicle growth and survival over long-term culture, follicles were cultured in growth media supplemented with or without 10 or 100 μg/mL LMW hyaluronan in a humidified environment of 5% CO_2_ in air at 37 °C for 12 days. Half of the growth media was changed every other day, and follicles were imaged at this time using the EVOS FL Auto with 10× and 20× objectives. To track individual follicles over time, follicles were identified by their relative positions. Images were used to measure average follicle diameters, which were calculated by averaging two perpendicular measurements taken from basement membrane to basement membrane using ImageJ software (National Institutes of Health, Bethesda, MD, USA). These measurements were plotted from day 0 until day 8 to obtain follicle growth curves. Measurements were not made past day 8, as some follicles merge together making it difficult to distinguish their basement membranes. Survival was assessed using pre-established morphological criteria [79]. Follicles were considered dead if they had an absent or unhealthy appearing oocyte and/or dark/pyknotic granulosa cells. In addition, follicles were also considered dead if any portion of their basement membrane was no longer intact. Survival data was plotted through the end of culture at day 12.

### 4.10. In Vitro Maturation and Meiotic Assessment

To assess the effect of LMW hyaluronan on oogenesis, in vitro maturation (IVM) was performed in treated and untreated follicles at 12 days of culture as previously described [79]. In brief, beads were incubated with 10 IU/mL alginate lyase (Sigma Aldrich, St. Louis, MO, USA) to release follicles from the alginate. Only morphologically healthy follicles that exhibited growth over the culture period and had an intact basement membrane at the end of culture were used for downstream analyses. Follicles were placed in αMEM Glutamax containing 5 ng/mL epidermal growth factor (EGF, GeneScript, Piscataway, NJ, USA), 0.2 mIU/mL FSH and 15 μg/mL hCG (Sigma Aldrich, St. Louis, MO, USA) for 19–20 h to mimic the luteinizing hormone (LH) surge. In vivo, this acute increase in LH levels triggers meiotic resumption and ovulation of oocytes from follicles. In a subset of samples, IVM was not performed and instead we manually recovered gametes from follicles on days 10 and 12 of culture after removal of the follicles from alginate with the alginate lyase. Follicles were transferred into 37 °C L15 containing 3 mg/mL polyvinylpyrrolidone (PVP) (Cat. # P2307, Sigma Aldrich, St. Louis, MO, USA) and 5 μL/mL Pen-Strep (L15/PVP/PS). Using a 100 μm stripper tip, gametes were extracted from follicles and immediately transferred into L15/PVP/PS with 0.025% milrinone (a PDE3 inhibitor that blocks meiotic resumption) and 0.3% (w/v) hyaluronidase. Following a brief incubation, cumulus cells were removed. All gametes were classified by morphological criteria (Supplemental Figure S2). First, gametes were categorized as normal or abnormal using transmitted light microscopy [80]. Next, the meiotic stage of all gametes classified as normal was assessed by light microscopy [79,81]. Finally, brightfield microscopy with an EVOS FL Auto microscope with 10X and 20X objectives was used to assess meiotic stage of gametes within intact follicles up until day 8 of culture. Meiotic stage was assessed using the criteria listed in Supplemental Figure S2.

### 4.11. Estradiol Assays of Conditioned Follicle Growth Media from eIVFG System

Concentrations of 17β-estradiol in the conditioned media from the eIVFG system were measured using ELISA kits (Calbiotech, Spring Valley, CA, USA) according to the manufacturer’s instructions. Conditioned media were collected from follicles grown with and without LMW hyaluronan treatment (50 μL/well per day) on day 0, 4, 8 and 12 of culture. Equal volumes of media were then pooled across replicates by day according to treatment group. Media collected from wells without follicles were used as a negative control. Assays were performed in triplicate for each experimental cohort.

### 4.12. Statistical Analysis

Data analysis was performed using R programming language and GraphPad Prism software (V7, GraphPad Software, La Jolla, CA, USA). In the cytokine protein array, differentially regulated proteins were defined as up-regulated (>1.45 fold-change relative to controls) or down-regulated (<0.55 fold-change relative to controls). For the real time PCR array, differentially expressed genes were defined as up-regulated (>1.45 fold-change relative to controls) or down-regulated (<0.55 fold-change relative to controls). To avoid Type II error (i.e., a false negative finding), statistical tests were not performed on cytokine array or qPCR data. A hypergeometric distribution test was used to test whether more eosinophil-related genes were differentially regulated following LMW hyaluronan treatment than would be expected by chance [34,35]. After normality was confirmed, t-tests were used to compare follicle growth and survival every other day of culture, as well as estradiol concentrations comparisons and meiotic progression comparisons between ovulated gametes from eIVFG cultures. All t-tests were two-tailed. A *p*-value of <0.05 was considered significant.

## Figures and Tables

**Table 1:** Differentially expressed inflammatory genes in ovarian stromal cells following 10 and 100 μg/mL LMW hyaluronan treatment.

Gene Type	Gene	Fold Change	
		10 μg/mL LMW-HA	100 μg/mL LMW-HA
Chemokines	*Ccl3*	1.75	
	*Ccl5*		1.61
	*Ccl6*	2.75	
	*Ccl8*	0.52	
	*Ccl12*	1.94	
	*Ccl19*	1.46	0.52
Chemokine Receptors	*Ccr3*	4.07	3.57
	*Ccr4*	1.53	
	*Ccr5*		0.19
	*Ccr6*		0.35
	*Ccr8*		0.32
	*Ccr10*	2.48	
	*Cx3cl1*	1.47	
	*Cxcl5*	2.19	
	*Cxcr3*	0.44	
Interleukins	*Il1* *α*		2.63
	*Il1B*	1.91	
	*Il4*		4.16
	*Il5*	0.42	0.02
	*Il15*		0.50
	*Il16*		0.52
Interleukin Receptors	*Il2rb*		0.51
Other Cytokines	*Fasl*		0.44
	*Lta*	0.18	
	*Ltb*		0.43
	*Tnfsf10*	0.41	
	*Tnfsf11*		0.27
Other Cytokine Receptors	*Tnfrsf11b*	1.46	

Fold changes are listed in green (up-regulation, >1.45 fold-change) or red (down-regulation, <0.55 fold-change).

## Supplementary Materials

Supplementary materials can be found at https://www.mdpi.com/1422-0067/21/3/1036/s1.

## Abbreviations

ECM	Extracellular matrix
DAMP	Damage-associated molecular signal
LMW	Low molecular weight
eIVFG	Encapsulated in vitro follicle growth
MII	Metaphase II
AP	Alkaline phosphatase
FSH	Follicle stimulating hormone
GV	Germinal vesicle
GVBD	Germinal vesicle breakdown
hCG	Human chorionic gonadotropin
