# Supplementary material for: Low Molecular Weight Hyaluronan Induces an Inflammatory Response in Ovarian Stromal Cells and Impairs Gamete Development In Vitro
Source: Int J Mol Sci. 2020 Feb 4;21(3):1036. doi: 10.3390/ijms21031036 (PMC7036885; doi:10.3390/ijms21031036)

Article

Low Molecular Weight Hyaluronan Induces an Inflammatory Response in Ovarian Stromal Cells and Impairs Gamete Development In Vitro

Supplemental Materials

Supplemental Table S1. List of 62 inflammatory cytokines profiled in cytokine array.

Cytokine
IL2
IL3
IL3 Rβ
IL4
IL5
IL6
IL9
IL10
IL-12 (p40/p70)
IL-12 (p70)
IL13
IL17A
CXCL1 (KC)
Leptin R
Leptin
CXCL5 (LIX)
CD62L (L-Selectin)
CXCL1 (Ltn)
MCP-1 (CCL2)
MCP-5
M-CSF
CXCL9 (MIG)
CCL3 (MIP-1 α)
MIP-1 γ
MIP-2
CCL19 (MIP-3 β)
CCL20 (MIP-3 α)
CXCL4 (PF-4)
P-Selectin
CCL5 (RANTES)
SCF
SDF-1 α
CCL17 (TARC)
TCA-3/CCL1 (I-309)
CCL25 (TECK)
TIMP-1
TNF-α
TNFRI (TNFRSF1A)

TNFRII (TNFRSF1B)
TPO
CD106 (VCAM-1)
VEGF-A

Supplemental Table S2. List of 84 inflammatory genes profiled in qPCR array and corresponding number key.

Gene Type	Gene	Number Key
Chemokines	*Ccl1*	1
	Ccl11	2
	Ccl12	3
	Ccl17	4
	Ccl19	5
	Ccl2	6
	Ccl20	7
	Ccl22	8
	Ccl24	9
	Ccl3	10
	Ccl4	11
	Ccl5	12
	Ccl6	13
	Ccl7	14
	Ccl8	15
	Ccl9	16
	Cx3cl1	17
	Cxcl1	18
	Cxcl10	19
	Cxcl11	20
	Cxcl12	21
	Cxcl13	22
	Cxcl15	23
	Cxcl5	24
	Cxcl9	25
Chemokine Receptors	*Ccr1*	26
	Ccr10	27
	Ccr2	28
	Ccr3	29
	Ccr4	30
	Ccr5	31
	Ccr6	32
	Ccr8	33
	Cxcr2	34
	Cxcr3	35
	Cxcr5	36
Interleukins	*Il11*	37
	Il13	38

	Il15	39
	Il16	40
	Il17a	41
	Il17b	42
	Il17f	43
	Il1a	44
	Il1b	45
	Il21	46
	Il27	47
	Il3	48
	Il33	49
	Il4	50
	Il5	51
	Il7	52
Interleukin Receptors	*Il10ra*	53
	Il10rb	54
	Il1r1	55
	Il1rn	56
	Il2rb	57
	Il2rg	58
	Il5ra	59
	Il6ra	60
	Il6st	61
Other Cytokines	*Aimp1*	62
	Bmp2	63
	Cd40lg	64
	Csf1	65
	Csf2	66
	Csf3	67
	Fasl	68
	Ifng	69
	Lta	70
	Ltb	71
	Mif	72
	Nampt	73
	Osm	74
	Pf4	75
	Spp1	76
	Tnf	77
	Tnfsf10	78
	Tnfsf11	79
	Tnfsf13	80
	Tnfsf13b	81
	Tnfsf4	82
	Vegfa	83
Other Cytokine Receptors	*Tnfrsf11b*	84

Supplemental Figure S1. Treatment with 100 μg/mL LMW hyaluronan differentially expresses 16 inflammatory genes. Using the $2^{-\Delta\Delta Ct}$ method, fold change in expression was calculated for each gene in treated over control samples. Gene expression fold changes for all 84 genes were $\log_2$ transformed to show expression changes relative to zero, and plotted by inflammatory gene type. Gene ty pes include "Chemokines", "Chemokine Receptors", "Interleukins", "Interleukin Receptors", "Other Cytokines" and "Other Cytokine Receptors". Up-regulation was defined as a fold change of 1.45 (equivalent to 0.54 on this graph) and down-regulation was defined as 0.55 (equivalent to -0.86 on this graph) were indicated with an asterisk (*). Grouped in this way, changes between treated and control samples can be qualitatively observed by gene function within an inflammatory response.

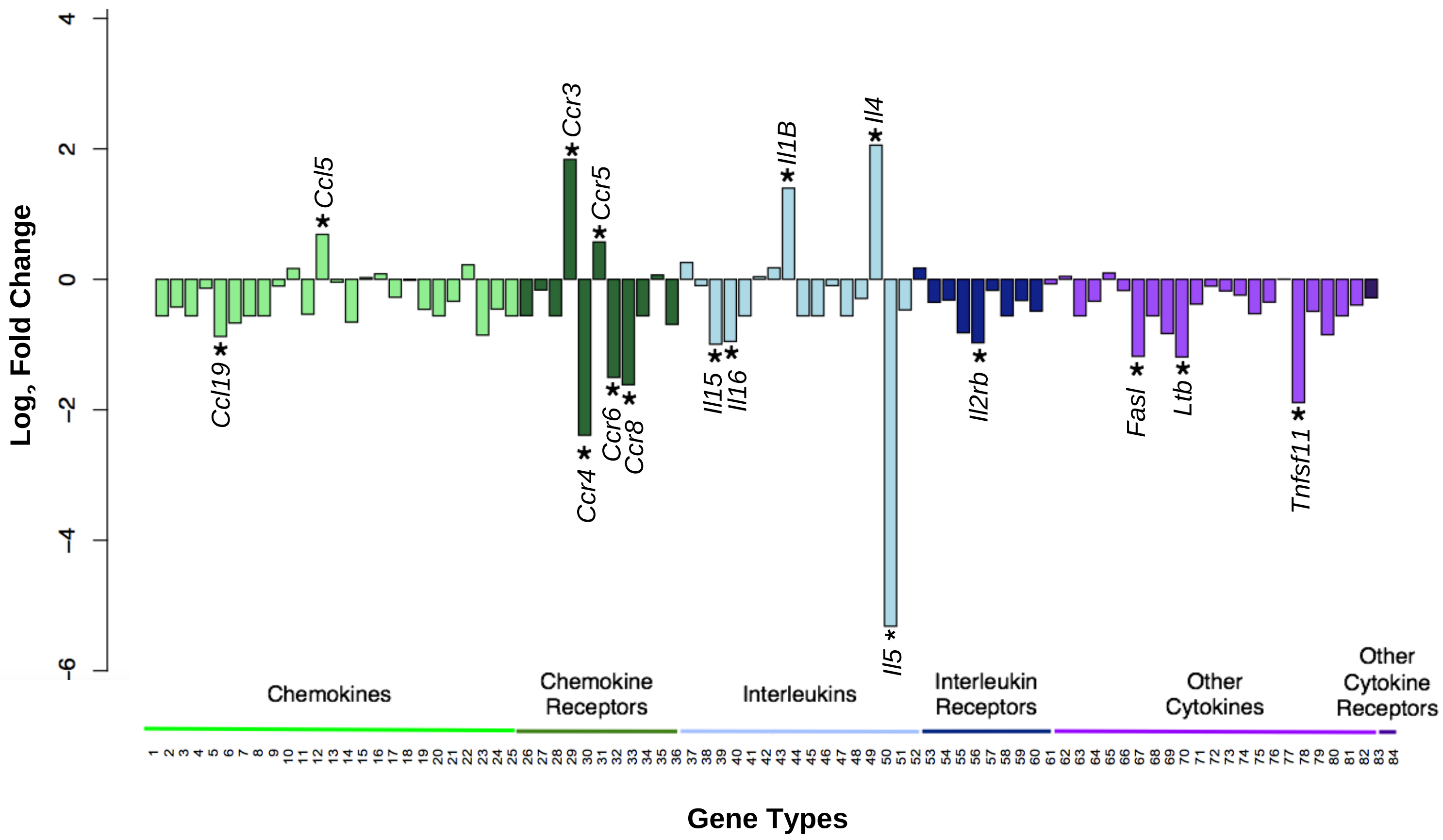

Supplemental Figure S2. Follicle culture and gamete quality and meiotic assessment in eIVFG system with and without *in vitro* maturation. Isolated secondary follicles were encapsulated in alginate beads and cultured in 10 µg/mL or 100 µg/mL LMW hyaluronan (LMW-HA). At the end of the culture period (either 10 or 12 days), healthy follicles were liberated from alginate beads and were removed from LMW hyaluronan-containing media for all downstream steps. To examine the impact of LMW hyaluronan on ovulated gametes following gonadotropin-induced meiotic resumption, healthy follicles were selected to undergo hCG-triggered *in vitro* maturation (IVM). Ovulated gametes were then collected and categorized as morphologically normal or abnormal based on pre-established criteria using brightfield microscopy. Any gametes fulfilling two or more of the following criteria were considered abnormal: large perivitelline space, retracted membrane, dark cytoplasm, or vacuoles and/or cytoplasmic inclusions. Any gamete that was degenerate was automatically categorized as abnormal. Next, meiotic stage was assessed in normal (not abnormal) gametes. Gametes that did not resume meiosis and remained arrested in prophase of meiosis I were identified by the presence of an intact germinal vesicle (GV) and were classified as GV-intact oocytes. Gametes that resumed meiosis and reached a mature metaphase-II (MII) stage were identified by the presence of an extruded polar body and categorized as MII eggs. Gametes without a polar body or a GV were in pro-metaphase I or metaphase I and defined as GV breakdown (GVBD). To examine whether LMW hyaluronan induced premature meiotic resumption in culture, gametes were directly harvested from follicles without any exposure to hCG (no IVM). Morphology and meiotic stage were determined using the same procedures listed above.

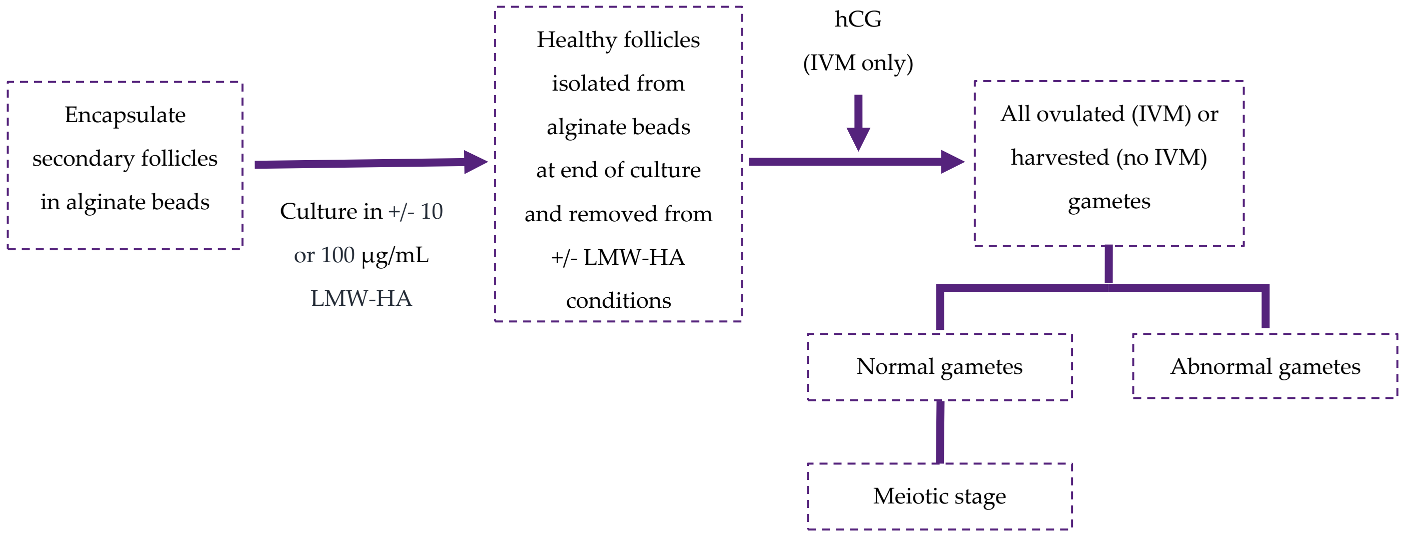

Supplement: Supplementary file 1 [file ijms-21-01036-s001.pdf]
